# Progress in Ultrasound Research on Non-Mass Breast Lesions: Definition, Classification, and Differential Diagnosis

**DOI:** 10.3390/diagnostics16081151

**Published:** 2026-04-13

**Authors:** Hui Wang, Jiaming Cui, Zexing Song, Mengwei Tao, Caiyun Niu, Chengpeng Zhao, Lihui Guo, Weiyang Zhang, Zhicheng He

**Affiliations:** 1 Department of Ultrasound, The First Hospital of Lanzhou University, Lanzhou 730000, China; niucy@lzu.edu.cn (C.N.); zhaochp@lzu.edu.cn (C.Z.); guolihui2021@163.com (L.G.); zwy624106@163.com (W.Z.); hezp@lzu.edu.cn (Z.H.); 2 The First Clinical Medical College, Lanzhou University, Lanzhou 730000, China; tsui_jm@126.com (J.C.); songzx2023@lzu.edu.cn (Z.S.); 18294226899@163.com (M.T.)

**Keywords:** non-mass breast lesion, ultrasound, BI-RADS, definition, classification, differential diagnosis

## Abstract

The objective of this review is to deepen the understanding and mastery of non-mass breast pathologies, enabling ultrasonographers to enhance diagnostic accuracy and improve their capabilities in image analysis and clinical interpretation. Narrative means have been used to synthesize evidence in this review. Currently, “non-mass breast lesions” are not included in ultrasound terminology of the 5th Edition Breast Imaging Reporting and Data System (BI-RADS). Although multiple classification systems have been proposed in the literature, there remains no standardized ultrasound definition or malignant risk grading for non-mass lesions. The ultrasound features of benign and malignant non-mass breast lesions are often subtle and partially overlapping, complicating differential diagnosis and impacting clinical evaluation and management. This paper reviews the ultrasound definitions and classifications of non-mass breast lesions, exploring the correlation between their ultrasound features and pathological histology as well as malignant risk. It also discusses the diagnostic values of conventional ultrasound, automated breast ultrasound, ultrasound elastography, and contrast-enhanced ultrasound for non-mass breast lesions. Finally, it compares the diagnostic accuracy of various ultrasound-guided needle biopsy techniques for non-mass lesions. Through the synthesis and summarization of the relevant literature, this paper aims to enhance the diagnostic proficiency of sonographers in evaluating non-mass breast lesions.

## 1. Introduction

Non-mass lesions in breast ultrasound refers to abnormalities exhibiting different echogenicity from surrounding tissues but failing to meet the criteria for a mass (i.e., a space-occupying lesion visible in at least two planes). The ultrasound terminology of the 5th Edition of the Breast Imaging Reporting and Data System (BI-RADS) includes masses, calcifications and associated features but lacks specific ultrasound terminology and malignancy classification criteria for “non-mass lesions” [1]. The literature reports indicate a 1.0–9.2% incidence of non-mass lesions in breast ultrasound screening [2,3,4], which may be attributed to the fact that non-mass breast lesions do not fall within the current appropriate use criteria of ultrasound for breast pathologies [5]. Due to the absence of standardized terminology for describing non-mass breast lesions, multiple descriptive terms have been used in the literature to define non-mass findings. In clinical practice, variations in sonographers’ recognition and interpretation of such lesions contribute to this diversity of definitions. More importantly, non-mass lesions on breast ultrasound encompass a broad spectrum of benign, benign with upgrade potential and malignant pathologies [6]. Therefore, accurate identification, proper analysis, and interpretation of non-mass lesions are crucial for enhancing the sensitivity and specificity of breast ultrasound screening.

This narrative review was conducted based on a comprehensive literature search. Electronic databases, including PubMed, Web of science, and CNKI, were searched from 2005 to 2025 to identify potential studies. The search strategy combined subject headings and text words, such as (“Non-mass breast lesion” OR “Ultrasound”) AND (“BI-RADS”). Studies were included if they (1) focused on the core topic of non-mass breast lesion; (2) were original clinical studies or high-quality reviews; and (3) were published in English or Chinese. Studies were excluded if they (1) were duplicated studies; (2) were irrelevant to the research focus; or (3) had incomplete data. Two reviewers independently conducted the initial screening based on titles and abstracts. Discrepancies were resolved by consensus or consultation with a third reviewer.

## 2. Definition and Classification of Non-Mass Breast Lesions

### 2.1. Ultrasound Definition of Non-Mass Breast Lesions

A breast mass refers to a nodular, space-occupying lesion formed by components differing from surrounding tissue. The studies employ various terms and descriptions for non-mass breast lesions. Park et al. and Giess et al. both consider non-mass breast lesions as those that do not exhibit the convex features of a mass in two orthogonal planes. In contrast, Lee et al. and Kim et al. both view non-mass breast lesions as distinct from the features of surrounding parenchymal tissue or the corresponding region of the contralateral breast. Additionally, Watanabe et al. and JSUM guidelines define non-mass lesions as lesions that cannot be identified as a mass. However, Ko et al. defines non-mass lesions as hypoechoic areas without an associated mass. All studies define non-mass lesions as ultrasound findings that do not conform to the shape of a mass (non-convex boundary) [2,3,7,8,9,10,11,12,13,14,15,16].

### 2.2. Ultrasound Classification of Non-Mass Breast Lesions

Currently, there is no widely accepted classification system for non-mass breast lesions, and classification systems proposed in the literature vary considerably (Table 1). In 2004, the Japan Association of Breast and Thyroid Sonology (JABTS) first proposed a classification system for non-mass breast lesions. This system categorizes non-mass breast lesions based on lesion distribution patterns and the presence or absence of calcifications. In 2005, the JABTS and the Japanese Society of Ultrasonics in Medicine (JSUM) jointly drafted diagnostic guidelines for non-mass breast lesions. The guidelines classify non-mass breast lesions as follows: ductal dilatation, ductal wall thickening, irregular ductal caliber, intraductal echoes or microcysts, polycystic changes, low echogenic areas in breast tissue, geographical low echogenic areas, low echogenic areas with blurred margins, and structural distortion [15]. The non-mass breast lesion classification system adopted by Kim et al. in 2014 was adapted from the guideline of the JABTS [2]. However, the scholars noted that ductal low echogenic areas overlap with ductal changes described in the BI-RADS, leading to their exclusion from this classification system.

In addition, Giess et al. classified non-mass breast lesions based on echo patterns and associated features, including hyperechoic halos, rear with acoustic shadow, calcifications, structural distortion, and ductal or tubular structures [7]. Wang et al. further subdivided non-mass lesions into hypoechoic areas, hypoechoic areas with microcalcifications, structural distortion, and solid hyperechoic lesions within ducts [9].

In addition, Lee et al. classified lesions based on echogenicity patterns into blurred, geographical, and mottled types, while also categorizing distribution into focal and regional patterns [3]. Meanwhile, Watanabe et al. followed the guideline of the JABTS and the JSUM, classifying non-mass lesions as ductal abnormalities, hypoechoic areas without distinct mass formation, structural distortion, multiple small cysts, and hyperechoic foci without hypoechoic areas [10].

Additionally, Ko et al. employed a classification method significantly different from previous approaches, categorizing non-mass lesions into four types: ductal hypoechoic lesions, hypoechoic areas without distinct masses, blurred regions with structural distortion, and ill-defined hypoechoic areas with acoustic shadowing [11]. Based on the positive predictive values of non-mass breast lesion subtypes outlined above, the research team categorized different non-mass breast lesion subtypes into BI-RADS categories 4A, 4B, and 4C. This classification system not only facilitates integration with BI-RADS but also helps clarify the indications for biopsy of non-mass breast lesions. Additionally, it was found that associated calcifications and the presence of abnormal axillary lymph nodes are significant predictors of malignant non-mass breast lesion. Park et al. categorized the distribution of non-mass lesions as focal, linear, or regional, with associated features including calcifications, architectural distortion, and ductal abnormalities [8]. Compared to studies by Ko et al., this study demonstrated better inter-observer consistency and identified more -significant predictors of malignant non-mass breast lesion, such as linear–segmental distribution and associated calcification or structural distortion, thereby enhancing the predictive ability for malignant non-mass breast lesion. Both Park et al.’s and Ko et al.’s studies employed a unified diagnostic standard to enhance inter-observer consistency, but neither conducted a Kappa analysis. Park et al. found that malignancy was significantly associated with palpability, and ultrasound features such as linear–segmental distribution, associated calcification, or structural distortion were significantly associated with malignant NML. In contrast, Ko et al.’s study only indicated that ultrasound-demonstrated associated calcification had a better correlation with malignant NML.

Similarly, Sotome et al. categorized non-mass lesions into four types: Type I: ductal dilatation with intraluminal echoes; Type II: polycystic changes; Type III: breast hypoechoic areas (without distinct mass formation), which is further subdivided into IIIa: speckled or mottled hypoechoic areas; IIIb: geographical hypoechoic areas; and IIIc: hypoechoic areas with indistinct margins [13]. This classification system overlaps significantly with the JABTS and JSUM guideline but is not entirely consistent. In 2023, the JSUM released its latest guideline for non-mass breast lesions, which synthesizes previous studies on the definition and classification of non-mass lesions, adopting a classification system based on echogenicity patterns combined with distribution patterns, which provides a comprehensive and precise definition of non-mass lesion classification, identifies typical ultrasound features corresponding to benign and malignant non-mass lesions, enhances the accuracy of diagnosing malignant non-mass lesions, and helps reduce breast cancer mortality [16].

In terms of the classification of non-mass breast lesions, the JSUM guideline, the Kim et al.’s classification, and the Lee et al.’s classification take into account both lesion type and lesion distribution patterns (diffuse and focal). However, the Kim et al.’s and Lee et al.’s classifications overlook the ductal abnormality type. Compared to other classifications, the Giess et al. and Park et al. classifications are relatively simple: Giess et al. classifies lesions based solely on echogenicity, while Park et al. classifies them based solely on distribution. Nevertheless, the relevant features proposed (including calcification, architectural distortion, and ductal abnormalities) to some extent compensate for the limitations of these classifications. Only the Lee et al. classification incorporates blood flow signals of lesions into classification; therefore, whether blood flow signals have classificatory value for non-mass breast lesions still needs to be confirmed by clinical practice. The Watanabe et al. classification defines lesion types similarly to the JSUM guideline but does not include lesion distribution patterns, which may lead to missed diagnoses of some non-mass breast lesions. Both the Ko et al. and Wang et al. classifications ignore multiple small cysts and have a narrower definition range for other types of non-mass breast lesions. Therefore, the JSUM guideline is currently the most comprehensive classification for non-mass breast lesions using routine ultrasound. Whether to include multimodal ultrasound information remains to be confirmed by clinical practice.

### 2.3. Ultrasonic Features of Non-Mass Breast Lesions

**Figure 1:**
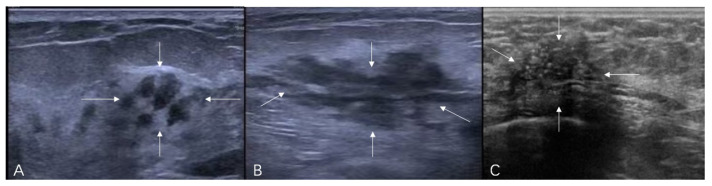
Ultrasound features of non-mass breast lesions of Type I. (**A**) A case of ductal carcinoma in situ (DCIS). Ultrasound shows patchy hypoechoic areas in the breast (indicated by the white arrows); (**B**) a case of DCIS. Ultrasound reveals mottled hypoechoic areas within the breast (indicated by the white arrows); (**C**) a case of DCIS. Ultrasound demonstrates ill-defined hypoechoic areas in the breast (indicated by the white arrows).

**Figure 2:**
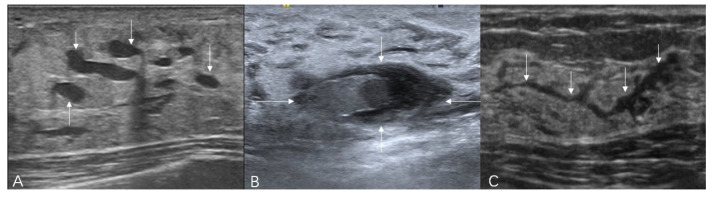
Ultrasound features of non-mass breast lesions of Type II. (**A**) Ultrasound shows dilated breast ducts, which may be a normal finding in late pregnancy and lactation (indicated by the white arrows); (**B**) a case of intraductal papilloma. Ultrasound reveals that the solid components within the dilated duct have a relatively regular shape (indicated by the white arrows); (**C**) a case of DCIS. Ultrasound shows irregular dilation of the breast ducts (indicated by the white arrows).

**Figure 3:**
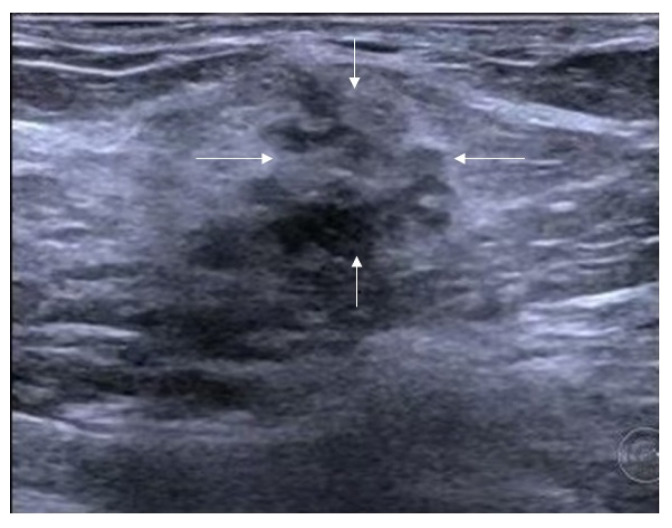
Ultrasound features of non-mass breast lesions of Type III. A case of DCIS. Ultrasound shows focal contraction/distortion of breast tissue at localized area within the breast (indicated by the white arrows).

**Figure 4:**
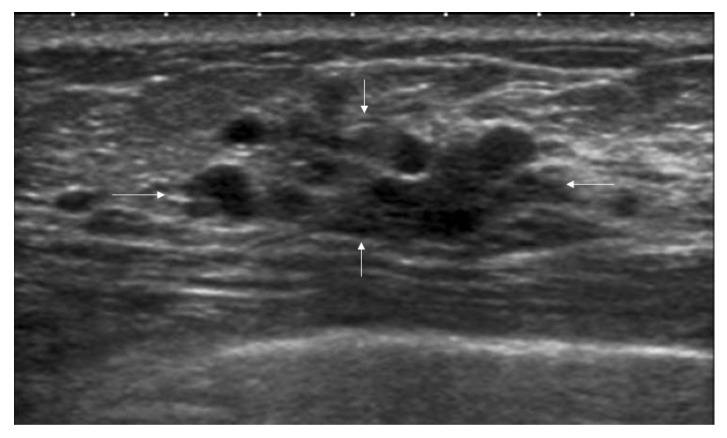
Ultrasound features of non-mass breast lesions of Type IV. A case of ductal papilloma. Ultrasound shows multiple small cysts interspersed with hypoechoic areas (indicated by the white arrows).

**Figure 5:**
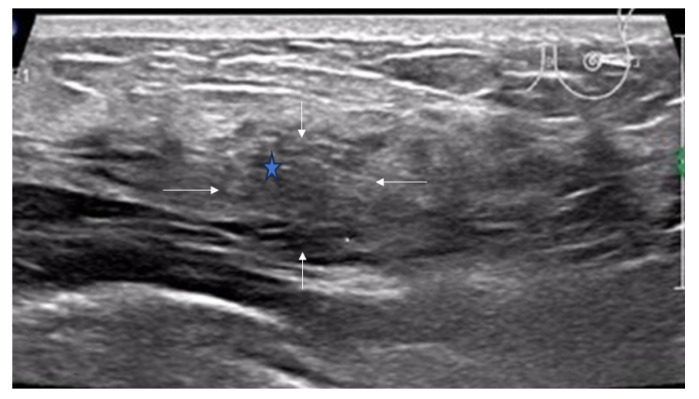
Ultrasound features of non-mass breast lesions of Type V. A case of DCIS. Ultrasound shows no obvious hypoechoic areas (indicated by the white arrows), only scattered hyperechoic foci (indicated by blue five-pointed star).

The ultrasound terminology of 5th Edition BI-RADS includes masses, calcifications, and related features (architectural distortion, ductal changes, skin changes, edema, vascular distribution, vascular grading, and elasticity assessment), but does not incorporate the concept of non-mass lesions [17,18]. It is hoped that the ultrasound terminology of the forthcoming 6th Edition BI-RADS will include non-mass lesions. Based on the literature’s definitions and classifications of non-mass breast lesions, combined with the authors’ clinical practice experience, the authors consider the latest guidelines published by the JSUM to be a relatively reasonable classification system. The latest classification system categorizes lesions based on their echogenicity patterns and distribution patterns. The echogenicity patterns include: Type I—intramammary hypoechoic areas (Figure 1), where Type Ia: patchy or mottled hypoechoic areas, i.e., multiple relatively small hypoechoic areas that can be recognized as a single lesion; Type Ib: geographical hypoechoic areas, where patchy/mottled hypoechoic areas merge; Type Ic: indistinct or ill-defined hypoechoic area, which cannot be recognized as patchy/mottled or geographical and cannot be identified as masses due to their indistinct or ill-defined borders; Type II—ductal abnormalities (Figure 2), where Type IIa: ductal dilatation, i.e., ducts markedly dilated beyond the areolar margin compared to adjacent ducts; Type IIb: ducts with internal echoes, which included three manifestations: solid echoes, i.e., visible hypoechoic components within ducts; echogenic foci, i.e., high-echo spots in the ducts; and floating echoes, i.e., visible breast milk, abscess, blood, or other fluid within ducts with observable flow; Type IIc: Irregularity of duct caliber; Type III—architectural distortion (Figure 3), i.e., manifestation of focal contraction/distortion of breast tissue at a specific point or localized area within the breast; Type IV—multiple small cysts measuring several millimeters observed within the breast tissue (Figure 4); Type V—echogenic foci without hypoechoic areas (Figure 5). The distribution patterns are classified as bilateral, unilateral, focal (clustered), segmental, or diffuse.

The latest JSUM guidelines and multiple scholars’ related research have identified the following ultrasound features suggesting malignant non-mass lesion: (1) hypoechoic areas with a linear or focal pattern in the breast and/or microcalcifications; (2) irregularity of the breast ducts; (3) architectural distortion; (4) abundant blood flow signals within and around the lesion [8,11,16]. Among these, hypoechoicity, microcalcifications, architectural distortion, and blood flow signals all fall within the ultrasound terminology of 5th Edition BI-RADS. In other words, although non-mass lesion is not included in the 5th Edition BI-RADS, the typical ultrasound features of malignant non-mass lesion can be used for BI-RADS classification. Currently, the ultrasound features of malignant non-mass lesion in the classification system are consistent with 5th Edition BI-RADS. However, the 5th Edition BI-RADS does not include the distribution pattern of breast lesions, which may lead to missed diagnoses of non-mass lesions.

Since NML is a special type of breast lesion, this study suggests that, in clinical practice, the management of such lesions should be defined and classified according to the latest JSUM guidelines, followed by malignant risk stratification using a modified BI-RADS, with the modified BI-RADS incorporating new malignant ultrasound features (such as irregular breast ducts) and adjusting the weights of existing malignant ultrasound features. This management protocol for non-mass lesion is proposed by the authors based on the current research status and findings, and it is believed to yield satisfactory outcomes.

### 2.4. Pathological and Histological Features of Non-Mass Breast Lesions

With increasing research on non-mass lesions and growing clinical emphasis on them, detection of such lesions has become more common. The detection rates and the proportion of benign and malignant non-mass lesions vary considerably among different institutions. Studies indicate that non-mass lesions account for 1.0–9.2% of breast diseases, with 46.2–90.6% being benign and 6.3–53.8% being malignant [3,4,8,9,19,20,21].

### 2.5. Pathological Tissue Composition of Non-Mass Breast Lesions

Lee et al. found that benign lesions, atypical lesions, and malignant lesions accounted for 90.6%, 3.1%, and 6.3% of non-mass lesions, respectively [3]. Wang et al. also reported that, among 80 non-mass lesions, benign lesions constituted approximately 46.2% and malignant lesions approximately 53.8% [9]. There were significant differences in the proportion of malignant non-mass lesions across the aforementioned studies. We analyzed that the reason may be due to different study populations. Lee et al.’s study population consisted of lesions that were mammographically negative but presented as non-mass lesions on ultrasound, whereas Wang et al.’s study population included lesions that presented as non-mass lesions on ultrasound. Malignant non-mass lesions primarily manifest as microcalcifications and architectural asymmetry on mammography. By excluding non-mass lesions with positive mammographic findings, Lee et al. largely reduced a substantial number of malignant non-mass lesions. Additionally, a report by Park’s team identified approximately 72.7% benign and 27.3% malignant lesions among 121 non-mass lesions [8]. Another large-scale study by the same team revealed 385 benign (53.8%) and 330 malignant (46.2%) lesions among 715 non-mass lesions [19]. Moreover, Kim et al.’s study of 139 non-mass lesions found benign lesions accounted for approximately 69.7%, while malignant lesions constituted about 30.2% [2]. Overall, detection rates for benign and malignant lesions remain inconsistent among studies. As sonographers deepen their understanding of non-mass lesions, detection rates for benign and malignant non-mass lesions may evolve.

### 2.6. Pathological Histological Types of Benign Non-Mass Breast Lesions

Several studies have reported the histological types of benign non-mass breast lesions, which include ductal papilloma, ductal epithelial hyperplasia, ductal ectasia, fibroadenomatous hyperplasia, fibrosis, radial scar, complex sclerosing lesions, sclerosing adenosis, mastitis, fibrocystic changes, mucinous cystic lesions, and diabetic mastopathy [3,8,9,11,19]. Furthermore, Wang et al. suggested that fibrocystic changes and adenosis are the most common benign non-mass lesions, while inflammatory breast disease is relatively rare [9].

### 2.7. Pathological Histological Types of Malignant Non-Mass Breast Lesions

**Figure 6:**
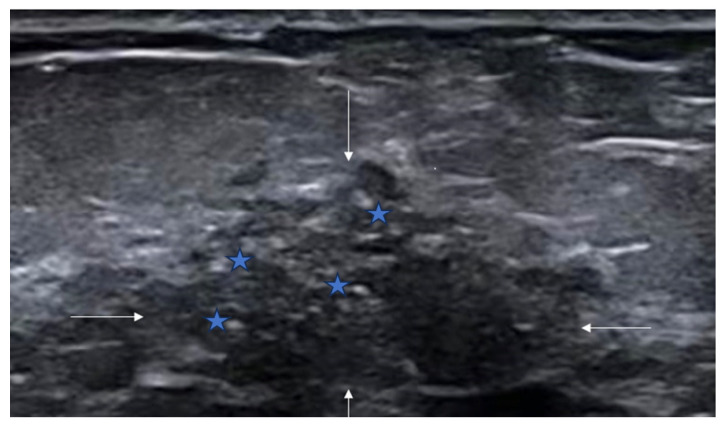
Ultrasound features of non-mass breast lesions of Type I. A case of invasive ductal carcinoma. Ultrasound reveals mottled hypoechoic areas within the breast (indicated by the white arrows), scattered hyperechoic foci (indicated by blue five-pointed star).

**Figure 7:**
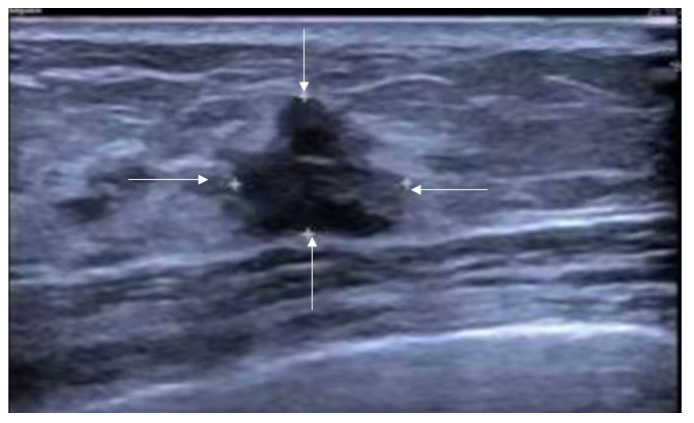
Ultrasound features of non-mass breast lesions of Type I. A case of invasive lobular carcinoma. Ultrasound demonstrates ill-defined hypoechoic areas in the breast (indicated by the white arrows).

The histological types of malignant non-mass breast lesions reported in the literature primarily include ductal carcinoma in situ, invasive ductal carcinoma with ductal components (Figure 6), invasive lobular carcinoma (ILC) (Figure 7), mucinous carcinoma, inflammatory carcinoma, breast lymphoma, and other invasive carcinomas [3,9,18,19,22,23,24]. Although invasive carcinoma predominates in non-mass lesions in some studies, Tsunoda et al. found ductal carcinoma in situ to be the primary histological type among malignant non-mass lesions classified by different ultrasound categories according to JABTS guidelines [18]. Meanwhile, Watanabe et al. reported that ductal carcinoma in situ accounted for approximately 60% of breast non-mass lesions [10]. More importantly, ductal carcinoma in situ frequently coexists with sclerosing lesions (including adenosis, radial scar, and complex sclerosing lesions), complicating the differential diagnosis between benign and malignant non-mass lesions.

### 2.8. Relationship Between Ultrasound Features of DCIS and Malignant Risk

DCIS, as the primary histological type of malignant non-mass breast lesions, has attracted the focus of scholars. Gunawardena et al. found that DCIS within non-mass breast lesions exhibits a 5-fold higher probability of high-grade lesions compared to mass lesions, a 7-fold higher probability of necrosis, and a 3-fold higher probability of calcification, which indicates that the ultrasound findings of non-mass breast lesions correlate with the histopathological features of high-grade DCIS [23]. In addition, Morita et al. observed that high-grade DCIS with extensive necrosis and necrotic calcifications is frequently associated with human epidermal growth factor receptor 2 (HER2) expression, while low-grade DCIS with secretory calcifications is typically linked to estrogen receptor expression [25,26]. Furthermore, Yao et al. also observed that DCIS with microinvasion is more likely to exhibit microcalcifications and rich vascularity than DCIS without microinvasion, and patients with this feature were more prone to sentinel lymph node metastasis, larger tumors, higher Ki-67 indices, and HER2 positivity [27]. The aforementioned study demonstrates that the ultrasound features of DCIS not only indicate hormone receptor expression in DCIS but also aid in distinguishing between “high-risk” and “low-risk” cases of DCIS.

## 3. Differential Diagnosis of Non-Mass Breast Lesions Utilizing Ultrasound

### 3.1. Diagnostic Value of BI-RADS for Non-Mass Breast Lesions

Although the concept of non-mass breast lesions has not been formally incorporated into the ultrasound terminology of the 5th Edition BI-RADS, some researchers have applied the BI-RADS to evaluate these lesions, which confirmed that the BI-RADS aids in stratifying the malignant risk of non-mass breast lesions [11,28]. Based on positive predictive values, Ko et al. categorized non-mass breast lesions into BI-RADS 4a (Type IIa), 4b (Types Ia, III, and IV), and 4c (Types Ib and IIb); compared to other types, Type Ib and IIb lesions exhibit higher malignant probability [11]. In addition, Lin et al. found that the BI-RADS demonstrated potential for diagnosing non-mass lesions with sensitivity (82.98%) and positive predictive value (84.78%); however, it underestimated the malignant risk for malignant non-mass lesions and overestimated the malignant risk for benign non-mass lesions [28]. Thus, distinct malignant risk stratification strategies should be applied for non-mass breast lesions and breast masses. More importantly, Uematsu et al. emphasized that understanding the clinical significance of non-mass lesions in breast ultrasound is crucial for patient management, since the current BI-RADS lacks a standardized definition for “non-mass breast lesions,” confusion persists in describing and managing such findings [29]. It is hoped that the 6th Edition BI-RADS will incorporate ultrasound terminology for describing non-mass breast lesions and a classification system for their malignant stratification.

### 3.2. The Diagnostic Value of ABUS for Non-Mass Breast Lesions

Automated breast ultrasound (ABUS) provides coronal plane information of breast lesions, serving as a valuable supplement to conventional ultrasound. Numerous studies have reported on ABUS’s role in the differential diagnosis of non-mass breast lesions [30,31,32]. Kwon et al. reported that ABUS is a highly visual technique for evaluating MRI-detected non-mass breast lesions, particularly suitable for malignant lesions [30]. Furthermore, Zhang et al. found that ABUS significantly improves sensitivity, specificity, and accuracy in diagnosing high-risk non-mass lesions detected by mammography, and demonstrated that ABUS’s coronal plane is more sensitive than conventional ultrasound in revealing architectural distortion [31]. The research team also found that ABUS outperformed mammography and conventional ultrasound in sensitivity, specificity, positive predictive value, negative predictive value, accuracy, and assessment of biopsy necessity for the differential diagnosis of non-mass breast lesions [32].

### 3.3. The Diagnostic Value of Ultrasound Elastography and Contrast-Enhanced Ultrasound for Non-Mass Breast Lesions

Ultrasound elastography and contrast-enhanced ultrasound (CEUS) provide multimodal ultrasound information about lesions and serve as important supplements to conventional ultrasound. Numerous reports have documented their value in the differential diagnosis of non-mass breast lesions [33,34,35,36,37,38,39]. Most scholars confirm that conventional ultrasound combined with ultrasound elastography and CEUS can significantly improve the differential diagnostic ability for non-mass breast lesions. Yang et al. demonstrated that combining BI-RADS with microvascular flow imaging, shear wave elastography, and CEUS yielded higher diagnostic sensitivity (95.24%), specificity (89.19%), positive predictive value (93.75%), negative predictive value (91.67%), and accuracy (93%) for diagnosing non-mass breast lesions compared to standalone methods [33]. Ko et al. also found shear wave elastography enhances the positive predictive value for BI-RADS 4a non-mass lesions, reducing unnecessary benign biopsies [34]. In addition, Cai et al. identified mean contrast signal intensity, perfusion rate, and 40 s enhancement intensity as the most effective dynamic parameters for diagnosing malignant non-mass breast lesions, achieving a diagnostic accuracy and sensitivity of 90.4% and 95.8%, respectively [35]. Furthermore, Zhang et al. demonstrated that CEUS enhancement intensity (*p* = 0.003), enhanced area (*p* = 0.005), and radial or penetrating vessels (*p* = 0.003) serve as independent diagnostic indicators for differentiating non-mass breast lesions, and the combined conventional ultrasound and CEUS demonstrated an area under the curve (AUC) of 0.885, a sensitivity of 94.6%, and a specificity of 77.8% [36]. Moreover, Li et al. confirmed that shear wave elastography combined with CEUS significantly enhances conventional ultrasound’s diagnostic value for non-mass breast lesions [37]. Guo et al. also reported that the combination of conventional ultrasound, ultrasound elastography, and CEUS achieve a sensitivity, specificity, positive predictive value, negative predictive value, accuracy, and AUC of 98%, 94%, 94.3%, 97.9%, 96%, and 0.96, respectively, for diagnosing non-mass breast lesions [39]. In contrast, Yin et al. concluded that multimodal ultrasound (conventional ultrasound + ultrasound elastography + CEUS) has limited value in differentiating non-mass breast lesions (AUC = 0.631), with relatively low sensitivity (65.6%) and specificity (81.2%) [38].

### 3.4. The Diagnostic Value of Artificial Intelligence-Assisted Ultrasound in Differentiating Non-Mass Breast Lesions

The medical–engineering intersection represents a significant developmental direction in ultrasound medicine, with artificial intelligence serving as the fundamental means to achieve this integration. Research on AI-assisted ultrasound for differentiating non-mass breast lesions has proliferated, with some developed models achieving diagnostic performance comparable to or exceeding that of highly experienced sonographers. Shi et al. demonstrated that integrating radiomics features with clinical ultrasound data significantly enhances diagnostic value for non-mass breast lesions (AUC: 0.93), aiding in distinguishing benign from malignant lesions [40]. In addition, Liu et al. demonstrated that a nomogram developed using radiomic features from intra-tumoral and peritumoral 2 mm regions in ultrasound images exhibits superior predictive performance for non-mass breast cancer (AUC: 0.98), with significant practical value [41]. Moreover, Wang et al. constructed a multimodal deep learning model utilizing grayscale and color Doppler ultrasound, demonstrating outstanding diagnostic efficacy in distinguishing benign from malignant non-mass breast lesions, and the model achieved 91.54% accuracy, 94.15% sensitivity, 87.30% specificity, and an AUC of 0.96, thereby aiding sonographers in evaluating non-mass breast lesions [42]. Meanwhile, Li et al. developed a deep learning model for classifying non-mass breast lesions based on ultrasound images, achieving good diagnostic performance on the test set with an AUC of 0.84, an accuracy of 70.5%, a sensitivity of 80.3%, and a specificity of 74.6%, which hold promise for improving the early diagnosis of non-mass breast lesions [43]. To address the scarcity of non-mass breast lesion cases, Hu et al. proposed a novel research framework that transfers the experience of deep learning for classifying breast masses to non-mass breast lesions, which improved the AUC for predicting malignant non-mass breast lesions by 10% compared to training directly on non-mass data [44].

### 3.5. Diagnostic Value of Ultrasound-Guided Needle Biopsy for Non-Mass Breast Lesions

Ultrasound-guided needle biopsy is an indispensable diagnostic method for non-mass breast lesions. Seo et al. compared the novel 13-gauge cordless vacuum-assisted biopsy (VAB) with the conventional 14-gauge semi-automatic core needle biopsy (CNB) for the ultrasound-guided biopsy of non-mass breast lesions [45]. Compared to CNB, VAB successfully and accurately biopsied non-mass breast lesions, with a significantly reduced missed diagnosis rate for non-mass lesions. Yashima et al. also performed ultrasound-guided biopsies of non-mass breast lesions using either a 16-gauge spring-loaded CNB or a 12-gauge spring-loaded VAB [46]. VAB demonstrated a significantly lower rate of upgrading from high-risk lesions to malignancy compared to CNB, confirming that ultrasound-guided VAB may be more suitable for needle biopsy of non-mass breast lesions.

## 4. Strengths and Limitations

The absence of standardized ultrasound terminology and criteria for assessing malignancy poses challenges for the clinical management of the non-mass breast lesions. The JSUM classification criteria categorize the ultrasound features of non-mass breast lesions into five subtypes, which can improve screening sensitivity of the non-mass breast lesions, though further validation with histopathological results is still required. Analyzing and summarizing the ultrasound features of benign, benign with upgrade potential and malignant non-mass breast lesions can enhance the accuracy of ultrasound diagnosis for malignant non-mass breast lesions. However, increased attention to non-mass breast lesions may lead to more false-positive cases in ultrasound screening, which is also a problem that cannot be ignored. The scientific management protocol for non-mass breast lesions proposed in this study also requires the combination of conventional ultrasound with advanced technologies (such as automated breast ultrasound, elastography, and contrast-enhanced ultrasound) to improve the differential diagnostic ability of benign and malignant non-mass breast lesions, thereby aiding clinical management decision-making.

## 5. Conclusions

The classification of non-mass breast lesions and the differential diagnosis between benign and malignant lesions contribute to the clinical diagnosis and management of non-mass breast pathologies.

## Figures and Tables

**Table 1:** Definitions and classifications of non-mass findings in the literatures.

Study	Definition	Classifications
Kim et al. [2]	A hypoechoic area whose feature was different from that of surrounding glands or the same area in the contralateral breast.	Non-mass breast lesion patterns: Mottled: Multiple small hypoechoic nodular tissue areas; Geographical: Fused hypoechoic regions without cobblestone appearance, resembling geographical map morphology; Blurred: Relatively homogeneous hypoechoic areas with indistinct borders. Non-mass breast lesion distribution: Focal distribution: Affecting less than one quadrant of the breast; Regional distribution: Affecting one or more quadrants of the breast.
Giess et al. [7]	Compared with the surrounding breast tissue, the orthogonal image shows identifiable areas of discrete echo texture changes, and their morphology does not conform to the convex features of a mass.	The echo patterns of non-mass lesions are classified as predominantly hypoechoic (>50%), predominantly hyperechoic, a mixture of hyperechoic and hypoechoic, or predominantly anechoic. Associated findings include: hyperechoic halo, acoustic shadow, calcification, structural distortion, or ductular or tubular structures.
Park et al. [8]	Lesions visible in two orthogonal planes cannot be characterized as a distinct mass due to the absence of clear margins or a defined shape.	Distribution of non-mass lesions: Focal: small, localized areas Linear–segmental: A longitudinal or triangular area arranged along a straight line or branches, involving one or more ducts. Regional: A large area of tissue that does not exhibit ductal or segmental distribution. Related features: calcification, structural distortion, and abnormal ductal changes.
Wang et al. [9]	Lesions that do not meet the strict criteria for masses on conventional ultrasound imaging.	Non-mass lesions are classified into the following categories: hypoechoic areas (regions with lower echogenicity), hypoechoic areas with scattered or diffuse microcalcifications, structural distortions (regions with disordered structures compared to normal tissue), and intraductal solid hyperechoic lesions (solid lesions within the ducts).
Lee et al. [3]	A hypoechoic area which does not conform to the definition of a “mass”, which is defined as a space-occupying lesion seen in two different planes and it has different character from that of the surrounding parenchyma or the same area in the contralateral breast.	The lesions were classified into indistinct, geographic, and mottled groups. An indistinct pattern was defined as a relatively uniform hypoechoic area whose margins were not clearly defined. A geographic pattern was defined as a confluent hypoechoic area with a cobblestone appearance including an aggregation of small, island-like hypoechoic areas. A mottled pattern was defined as a number of small, island-like hypoechoic areas in the mammary parenchyma. The lesion distribution classified into focal and regional groups. A focal distribution was defined as a lesion which was scanned within the field-of-view technology of the linear probe. A regional distribution was defined as a lesion which exceeded the field-of-view technology and needed the use of extended field-of-view technology. The internal vascularity of the lesions on power Doppler images was classified into absent and present groups.
Watanabe et al. [10]	Non-mass abnormalities are defined as lesions that are not recognized as a mass.	Non-mass abnormalities were classified into five groups. Duct abnormalities usually refer to ductal dilation accompanied by filling of the duct with a solid component. Hypoechoic areas in the mammary gland differ from the surrounding tissue and cannot be recognized as masses. Architectural distortion is a term describing a lesion that distorts the breast tissue, but without mass formation. Multiple small cysts are defined as multiple tiny or small cysts existing in the mammary gland. Echogenic foci without a hypoechoic area are lesions in which only microcalcifications are visible.
Ko et al. [11]	Non-mass lesions are defined as a hypoechoic area without an associated mass.	Non-mass lesion patterns were classified into four types: Type I, a ductal hypoechoic area has duct-like structures with parallel orientation. Type Ia: no associated echogenic dots representing calcifications in a ductal hypoechoic area. Type Ib: associated internal calcifications were present in a ductal hypoechoic area. Type II, a non-ductal hypoechoic area is visible as a confined asymmetry with an indistinct shape on two different projections that does not form a definite mass and that differs from the surrounding glandular tissue or the same area in the contralateral breast. Type IIa: no associated internal calcifications were identified in a non-ductal hypoechoic area. Type IIb: associated internal calcifications were present in a non-ductal hypoechoic area. Type III, a vague area of altered echotexture with associated architectural distortion is observed. Type IV, an indistinct hypoechoic area with associated posterior acoustic shadowing is present.
Ito et al. [16]	Non-mass abnormalities refer to lesions that are difficult to discern as masses on US images.	Non-mass lesion patterns were classified into five types: Type I: Hypoechoic area in the mammary gland Type Ia: Patchy or mottled hypoechoic area Type Ib: Geographic hypoechoic area Type Ic: Indistinct or ill-defined hypoechoic area Type II: Abnormalities of the ducts Type IIa: Duct dilatation Type IIb: Ducts with internal echoes Solid echoes; Echogenic foci; Floating echoes Type IIc: Irregularity of ductal caliber Type III: Architectural distortion Type IV: Multiple small cysts Type V: Echogenic foci without a hypoechoic area Lesion distribution patterns: Bilateral, unilateral; Focal (clustered), segmental, diffuse

## Data Availability

No new data were created or analyzed in this study. Data sharing is not applicable to this article.
